# Performance Assessment of Four Chimeric *Trypanosoma cruzi* Antigens Based on Antigen-Antibody Detection for Diagnosis of Chronic Chagas Disease

**DOI:** 10.1371/journal.pone.0161100

**Published:** 2016-08-12

**Authors:** Fred Luciano Neves Santos, Paola Alejandra Fiorani Celedon, Nilson Ivo Tonin Zanchin, Tatiana de Arruda Campos Brasil, Leonardo Foti, Wayner Vieira de Souza, Edmilson Domingos Silva, Yara de Miranda Gomes, Marco Aurélio Krieger

**Affiliations:** 1 Aggeu Magalhães Research Center, FIOCRUZ-PE, Recife, PE, Brazil; 2 Molecular Biology Institute of Paraná, Curitiba, PR, Brazil; 3 Carlos Chagas Institute, FIOCRUZ-PR, Curitiba, PR, Brazil; 4 Biomanguinhos, FIOCRUZ-RJ, Rio de Janeiro, RJ, Brazil; Universidade Federal de Pelotas, BRAZIL

## Abstract

The performance of serologic tests in chronic Chagas disease diagnosis largely depends on the type and quality of the antigen preparations that are used for detection of anti-*Trypanosoma cruzi* antibodies. Whole-cell *T*. *cruzi* extracts or recombinant proteins have shown variation in the performance and cross-reactivity. Synthetic chimeric proteins comprising fragments of repetitive amino acids of several different proteins have been shown to improve assay performances to detect Chagasic infections. Here, we describe the production of four chimeric *T*. *cruzi* proteins and the assessment of their performance for diagnostic purposes. Circular Dichroism spectra indicated the absence of well-defined secondary structures, while polydispersity evaluated by Dynamic Light Scattering revealed only minor aggregates in 50 mM carbonate-bicarbonate (pH 9.6), demonstrating that it is an appropriate buffering system for sensitizing microplates. Serum samples from *T*. *cruzi*-infected and non-infected individuals were used to assess the performance of these antigens for detecting antibodies against *T*. *cruzi*, using both enzyme-linked immunosorbent assay and a liquid bead array platform. Performance parameters (AUC, sensitivity, specificity, accuracy and J index) showed high diagnostic accuracy for all chimeric proteins for detection of specific anti-*T*. *cruzi* antibodies and differentiated seropositive individuals from those who were seronegative. Our data suggest that these four chimeric proteins are eligible for phase II studies.

## Introduction

Chagas disease (CD) is a potentially life-threatening zoonosis caused by the hemoprotozoan parasite *Trypanosoma cruzi*, which may be transmitted by contact with feces/urine of infected blood-sucking triatomine bugs, consumption of contaminated food or beverages, blood transfusion, tissue and organ transplantation, from mother-to-child during pregnancy, and laboratory accidents [1]. *T*. *cruzi* infections are found mainly in endemic areas of the 21 Latin American countries and is responsible for the highest estimated global burden of the neglected tropical diseases (NTD), causing mortality in 14,000 people annually and morbidity in up to 8 million people [2]. In the past few decades, *T*. *cruzi* infection has been increasingly detected beyond Latin America and has become an infection of worldwide health concern due to infected people migrating to many European and Western Pacific countries, the United States, and Canada [3,4].

The new scenario of worldwide distribution of CD potentiates the risk for blood and organ banks and donors. Strict screening measures should therefore be in place, because chronic/indeterminate forms of CD do not show any specific symptoms. Diagnostic methods for detecting *T*. *cruzi* infection need to consider both clinical symptoms as well as the stage of illness. In the initial or so-called “acute” phase, the parasite can be easily detected in blood smears owing to high levels of parasitemia. At this stage, PCR-based methods can be adopted as efficient diagnostic tools, because specific antibodies can only be found several weeks after infection. In lifelong chronic infections, parasitemia is low but high levels of specific anti-*T*. *cruzi* antibodies (IgG) can be found in patient’s blood [5]. In chronic infections, CD diagnosis requires the use of antigen-antibody detection methods, which are carried out by immunological techniques.

Several immunological tests and methods based on different technological platforms are available for CD diagnosis. These tests utilize traditional enzyme-linked immunosorbent assay (ELISA), indirect hemagglutination, immunofluorescence antibody [6], flow cytometry [7] as well as optical reader-based methods such as liquid microarray (LMA) [8], among others. However, the performance of these tests is dependent on the antigen preparations used to detect the anti-*T*. *cruzi* antibodies [9]. Earlier versions of serological tests used whole extracts of the non-invasive epimastigote forms of *T*. *cruzi*, because this is the safest parasite stage, which is also the most cost-efficient to culture. The Brazilian Health Ministry recommends tests based on whole cell lysate as antigens; however, combinations with more specific assays are also advised for greater reliability [6]. Two assays with different platforms for CD diagnosis are also recommended by the WHO [10]. However, the accuracy of these assays is often challenged due to false-positives and inconclusive results that can occur especially in the case of cross-reactivity with related protozoan parasites, particularly *Leishmania* spp. and *Trypanosoma rangeli* [11,12].

In Brazil, epidemiological approaches to control vectorial transmission of CD by health Institutions have been effective in the past decades. Nevertheless, new oral infection cases, prevalence of around 2 million chronically infected individuals and the coexistence of other parasites in the same geographical areas, mainly the northeastern region, demand permanent efforts to ensure the monitoring and diagnosis under strict control [13]. Therefore, cross-reactivity and inconclusive results have boosted efforts to develop the production of more specific and more sensitive tests. An important point is to obtain high specificity for local infections, taking into account the genetic and biological variability among *T*. *cruzi* isolates [14,15] that may correlate with an individual’s immunological response, which can influence the efficiency of immunodiagnostic tests.

Antigens for efficient detection of IgG antibodies must contain the most immunogenic sequences of the microorganism. The short repetitive sequences of amino acids in *T*. *cruzi* are known to be highly reactive epitopes [16,17], although non-repetitive antigens can also be recognized by serum antibodies [18]. An important factor for increasing test reactivity appears to be the presence of a minimum number of repetitive epitopes [19]. Using ELISA, Hernández et al [20] showed that chimeric constructs could perform better than mixtures of the same isolated sequences. Similar results were reported by Camussone et al [21] suggesting that multiepitope antigens might render a greater available epitope-to-well active site ratio, once adsorption to the well-binding sites blocks only part of the active regions, leaving the rest of the specific sites free to interact with the serum antibodies. This blockage would be more severe for short sequences that could lose the antibody-specific region during the adsorption to the plate well.

In this study, previously well-characterized *T*. *cruzi* sequences were selected from the literature and four chimeric constructs were optimized and synthesized for recombinant production in *Escherichia coli*. The study aimed to pre-evaluate the performance of these four recombinant chimeric antigens in two robust technological platforms (ELISA and LMA) for CD diagnosis of human sera from an endemic region in Brazil. All sera were tested using two commercial kits selected accordingly Santos et al (2016). The antigen sequences were selected from the literature using their previous use in ELISA as inclusion criteria, radioimmunoprecipitation assay (RIPA), or lateral flow tests of populations from different regions [22–24], subcellular localization (cytoskeleton and membrane associated, cytoplasmic and shed proteins) [25,26], tandem repeats, presence in chronic infective phase [27,28], low cross-reactivity with other protozoans [29–31], among other criteria.

## Materials and Methods

### Ethical considerations

This investigation was approved by the Ethical Committee for Human Research from Aggeu Magalhães Research Center, Oswaldo Cruz Foundation, Recife-PE (CAEE: 15812213.8.0000.5190), following the principles of the Declaration of Helsinki. We used samples from the biorepository of the Reference Laboratory for Chagas Disease. In order to protect the patients private information the Ethical Committee approved that the samples were anonymized so that the researchers do not have access to patient’s private information therefore avoiding the need of verbal or written consent.

### Acquisition of synthetic genes, protein expression and purification

Optimized synthetic genes for *E*. *coli* expression of the four *T*. *cruzi* chimeric antigens (named IBMP8-1, IBMP8-2, IBMP8-3 and IBMP8-4) were acquired from a commercial supplier (GenScript, Piscataway-NJ, USA). The synthetic genes purchased in the pUC57 were subcloned in-house into the pET28a *E*. *coli* expression vector. The genes encode proteins with predicted molecular weight of 17, 36, 30 and 45 kDa, respectively.

Recombinant antigens were expressed in *Escherichia coli* BL21-Star (DE3), grown in LB medium supplemented with 0.5 M of isopropyl β-D-1-thiogalactopyranoside (IPTG). The antigens were purified by two chromatographic steps as follows: affinity chromatography, buffer exchange/desalting/concentration by ultrafiltration and ionic exchange chromatography. Alternatively, the last step was affinity chromatography with a different binder. Pure proteins were quantified by a fluorimetric assay (Qubit® 2.0, Invitrogen Technologies, Carlsbad-CA, USA). Expression and purity of the recombinant antigens were checked by SDS-PAGE [32]. After protein induction 7 μl of the total bacterial proteins were separated by electrophoresis and polyacrylamide gels were stained with CBB-G250.

### Secondary structural analysis and aggregation profile

The stability and polydispersity of the IBMP chimeric recombinant proteins were assessed by adopting three buffering systems: 50 mM carbonate-bicarbonate, pH 9.6; 50 mM sodium phosphate, pH 7.5; and 50 mM MES ([2-(n-morpholino) ethanesulfonic acid], pH 5.5).

### Circular dichroism spectroscopy

Secondary structure of the proteins was recorded by far-UV circular dichroism spectra at room temperature (RT) on a Jasco J-815 spectropolarimeter (Jasco, Tokyo, Japan). All samples were analyzed at 0.2 mg/ml in a 1-mm path length quartz cuvette. A total of four scans were acquired in continuous mode, corrected by subtracting the spectrum of the buffering system under the same conditions and then averaged for final analysis. Each scan in the range of 200–260 nm was obtained with scanning rate of 100 nm/minute, 1 nm bandwidth, data integration time of 1 s, and 0.5 nm data pitch.

### Dynamic light scattering (DLS)

IBMP recombinant chimeric protein aggregation profile was investigated by DLS. The measurements were performed on a Dynamic Light Scattering DynaPro NanoStar (Wyatt Technology Corp., Santa Barbara-CA, USA), equipped with a Ga-As laser (120 mW) operating at a nominal wavelength of 658 nm. Protein samples were placed in 10 mm diameter glass tubes, and measurements performed at RT. Data analysis was performed using Dynamics version 7.1.7.16.

### Sample collection

Anonymized human serum samples from individuals either non-infected (n = 20) or infected (n = 280) with *T*. *cruzi* from different endemic areas for CD in Pernambuco (Brazil) were obtained from the Reference Laboratory for Chagas Disease (RLCD, Oswaldo Cruz Foundation/PE, Brazil) and used to compare the performance of four IBMP chimeric proteins for *T*. *cruzi* by antigen-antibody detection. Sample selection was based on positivity or negativity by two serological tests for CD: Imuno-ELISA Chagas (Wama Diagnóstica, São Palo, Brazil, batch 14D061), which is based on recombinant antigens; and ELISA Chagas III (BIOSChile, Ingeniería Genética S.A., Santiago, Chile, batch 1F130525), which uses whole extracts of *T*. *cruzi* strains Mn and Tulahuen as antigens [33]. Samples with disagreeing results between both tests or judged to be inconclusive in one of them were excluded. Each sample was given an identifier code in the laboratory to ensure a blinded analysis.

### Laboratory assays

The presence of specific antibodies in human serum samples against the IBMP recombinant chimeric proteins (-8.1, -8.2, -8.3 and -8.4) was evaluated using in-house ELISA and LMA in order to assess the capability of these proteins to differentiate efficiently, positive from negative samples.

#### In-house ELISA optimization and procedure

The optimal dilutions of serum and antibody-enzyme (horseradish peroxidase; HRP) conjugate were determined by checkerboard-titration using *T*. *cruzi* antigens at different concentrations. With an aim to reduce nonspecific binding between the microplate and unrelated proteins, fetal bovine serum (FBS) and bovine serum albumin (BSA) were evaluated as blocking agents. The final conditions were chosen on the basis of the maximum contrast in the average optical density (OD) value between positive and negative samples plus 3 standard deviations (SD). The results were considered acceptable when positive samples had average OD above 1.0 and negative samples below 0.2. After optimization, IBMP chimeras were used at 12.5 ng (IBMP-8.2) and 25.0 ng per well (IBMP-8.1, -8.3 and -8.4) in coating buffer (0.05 M carbonate-bicarbonate, pH 9.6) in transparent “Maxisorp” 96-well microplates (Nunc, Roskilde, Denmark). Microplates were blocked with Well Champion reagent (Kem-En-Tec, Taastrup, Denmark) according to the manufacturer’s instructions. Serum samples (100 μl) were loaded at 1:100 in 0.05 M phosphate-buffered saline (pH 7.2)-0.5% Tween 20 (PBS-T) for IBMP-8.2-, -8.3- and -8.4-coated plates and in PBS-T-0.5% FBS for IBMP-8.1-coated plates and incubated at 37°C for 60 min. Following incubation, the microplates were washed in wash buffer (PBS-T) using an automated washer to remove unbound antibodies. HRP conjugated goat anti-human IgG (Biomanguinhos, FIOCRUZ/RJ, Brazil, batch 135EXCJAP0047) was diluted 1:40,000 in PBS-T, and 100 μl were then added to each well and the microplates were incubated for 30 min at 37°C. After five new washes, the immune complexes were revealed by the addition of 100 μl TBM substrate (tetramethyl-benzidine; Kem-En-Tec, Taastrup, Denmark). After 15 min incubation at RT in the dark, the reaction was stopped with 50 μl 5 N H_2_SO_4_, and the absorbance at 450 nm was measured in a Multiskan® FC microplate spectrophotometer (Thermo Scientific^TM^, Finland).

#### IBMP antigen coupling to microsphere and LMA procedure

The antigens were coupled under different conditions of buffer pH and antigen concentration: IBMP-8.1 in PBS pH 7.4 at 66 μg/ml, IBMP-8.2 in PBS pH 7.4 at 80.3 μg/ml, IBMP-8.3 in PBS pH 7.2 at 70 μg/ml and IBMP-8.4 in PBS pH 7.0 at 70 μg/ml. These conditions correspond to the higher signal to noise ratios and the best ROC curve after assaying them against positive and negative serum samples. Coupling of IBMP antigens to paramagnetic carboxylated microspheres (Luminex Corp, Austin-TX, USA) was performed using the manufacturer’s protocol. Briefly, a suspension of 2 x 10^6^ microspheres was mixed by ultrasound bath (Cole-Parmer ultrasonic cleaner, Cole-Parmer Instruments Company, Vernon Hills-IL, USA) and horizontal agitation (IKA vortex genius 3 VG3S32, IKA do Brasil, Campinas-SP, Brazil) to ensure homogeneous distribution of the suspension. After two washes, the microspheres were suspended in 400 μl of activation buffer (100 mM sodium phosphate, pH 6.3). Solutions (50 μl of each) of N-hydroxysulfosuccinimide (Pierce, Rockford-IL, USA) and 1-ethyl-3(3-dimethylaminopropyl)-carbodiimide hydrochloride (Pierce), both diluted to 50 mg/ml in double-distilled water (dH_2_O), were added to chemically activate the microspheres. After mixing, the microspheres were incubated for 20 minutes in the dark at 25°C at 250 rpm lateral agitation. The activated microspheres were subsequently washed twice with coupling buffer, after which 200 μl of antigen was diluted in the coupling buffer at the chosen concentration. These suspensions were incubated at 250 rpm horizontal agitation for 2 hours at 37°C. After incubation, the microspheres were washed three times with washing buffer (PBS, containing 1% BSA, 0.05% Tween 20). The final microsphere suspensions were counted (Beckman Coulter Z3, Kendall-FL, USA) and adjusted to a concentration of 4 x 10^4^ microspheres/ml in storage buffer (PBS containing 1% BSA and 0.02% sodium azide) and stored, protected from light at 2–8°C in low binding tubes (#0030 108.116, Eppendorf, Hamburg, Germany) for 24 hours. The LMA immunoassays were performed using serum samples diluted to 1:200 in assay buffer (PBS containing 1% BSA, 0.05% Tween 20). Fifty μl of microsphere suspension (~2,500) and 50 μl of diluted serum were mixed in each well of a 96-well plate and incubated for 15 min in the dark at 37°C with horizontal rotation at 600 rpm. The microspheres were then washed twice with 100 μl of wash buffer in Hydroflex plate washer with a magnetic plate support (TECAN, Durham-NC, USA). Goat anti-human IgG conjugated to phycoerythrin (GTIG-001, Moss substrates, Pasadena-MD, USA) diluted 1:1,000 in assay buffer was added and microspheres were incubated for 15 min in the dark at 37°C with horizontal rotation at 600 rpm. The microspheres were washed twice with 200 μl of wash buffer and once with Sheath Fluid 1x (Luminex Corp, Austin-TX, USA). The results, expressed as median fluorescence intensity (MFI), was determined with a Luminex 200 device.

#### Statistical analysis

Data were encoded and analyzed using scatter computer graphic software (GraphPad Prism version 6, San Diego-CA, USA). Descriptive statistics were presented as geometric mean ± SD. To test the normality of datasets, the Shapiro-Wilk test followed by Student’s t-test was used, and when variance homogeneity assumption was not confirmed, the Wilcoxon signed-ranks test was used. All analyses were two-tailed and a *p*-value less than 5% was considered significant (*p* < 0.05). Cut-off point analysis was used to identify the optimal value of OD for ELISA and MFI for LMA that differentiates negative from positive samples. The threshold was defined by the largest distance from the diagonal line of the receiver operating characteristic curve (ROC) (sensitivity x (1-specificity)). The results were expressed by plotting as an index that represents the ratio between the OD (EIA) or MFI (LMA) of the samples and the OD (EIA) or MFI (LMA) of the cut-off. This index is referred to as reactivity index (RI) and all results < 1.00 were considered negative. However, samples were deemed inconclusive (or in gray zone) if the RI values fell into the undetermined zone, which was hypothesized as RI values of 1.0 ± 10%. The ELISA and liquid bead microarray test performances were computed using a dichotomous approach and compared in terms of sensitivity (Se), specificity (Sp), accuracy, and Youden index (J) [34]. A confidence Interval (CI) was constructed to address precision of the proportion estimates with a confidence level of 95%. The strength of agreement with ELISA was assessed by the Cohen’s Kappa coefficient (κ) [35], which accounts for agreement taking place only by chance beyond simple percent agreement calculations. Its values are interpreted as poor (*κ* ≤ 0), slight (0 < *κ* ≤ 0.20), fair (0.21 < *κ* ≤ 0.40), moderate (0.41 < *κ* ≤ 0.60), substantial (0.61 < *κ* ≤ 0.80) and almost perfect agreement (0.81 < *κ* ≤ 1.0).

## Results

### Acquisition of recombinant chimeric proteins

**Fig 1:**
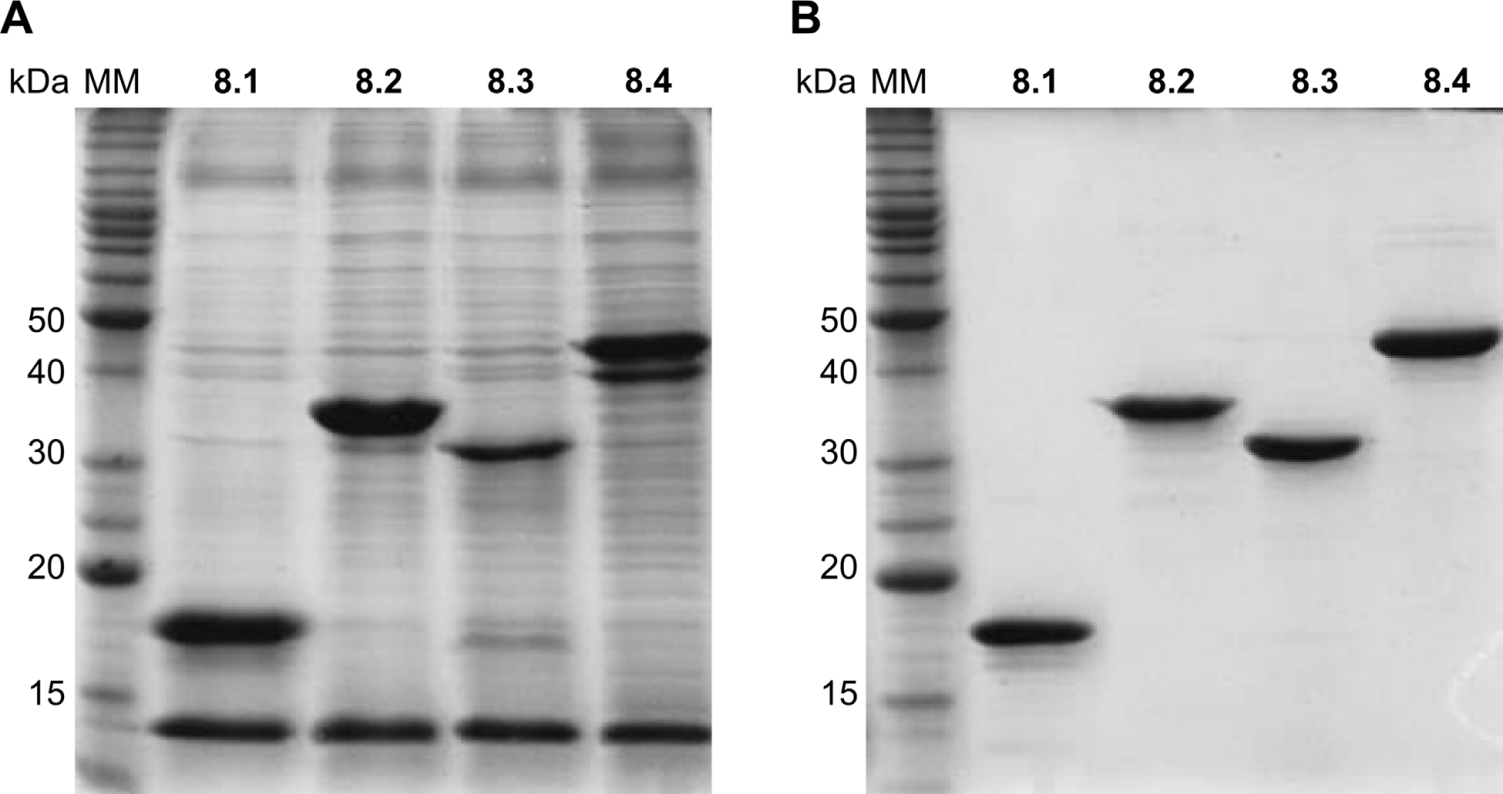
SDS-polyacrylamide gel stained with Coomassie Brilliant Blue G-250. IBMP chimeric proteins were purified from the total protein extracts (A) and 1.5 μg of each pure recombinant antigen were applied per lane (B). MM: molecular weight marker. IBMP-8.1 (17 kDa); IBMP-8.2 (36 kDa); IBMP-8.3 (30 kDa); IBMP-8.4 (45 kDa).

After cell-disruption and centrifugation, recombinant proteins were recovered from the supernatant and the purity was estimated to be over 95% (Fig 1). It was not possible to obtain proteins with satisfactory purity with one-step purification; therefore, two chromatographic steps were necessary as described. Satisfactory recovery yield was ranged from 3 to 5 mg/l of the culture volume, IBMP 8–3 being the least productive.

### Secondary structural analysis and aggregation profile

**Fig 2:**
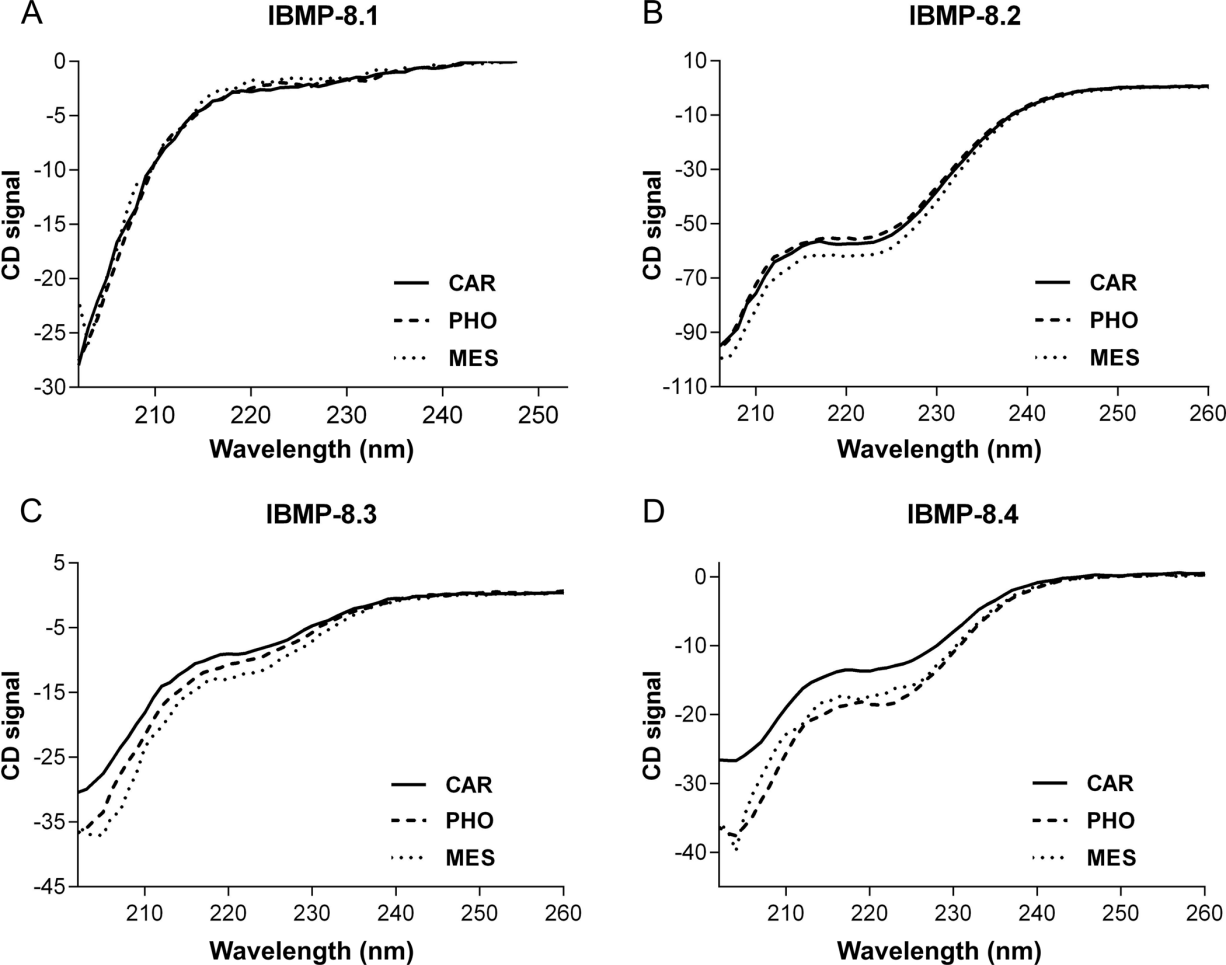
Far-UV circular dichroism spectra for IBMP recombinant chimeric proteins. Panel A) IBMP-8.1; Panel B) IBMP-8.2; Panel C) IBMP-8.3; Panel D) IBMP-8.4.

**Fig 3:**
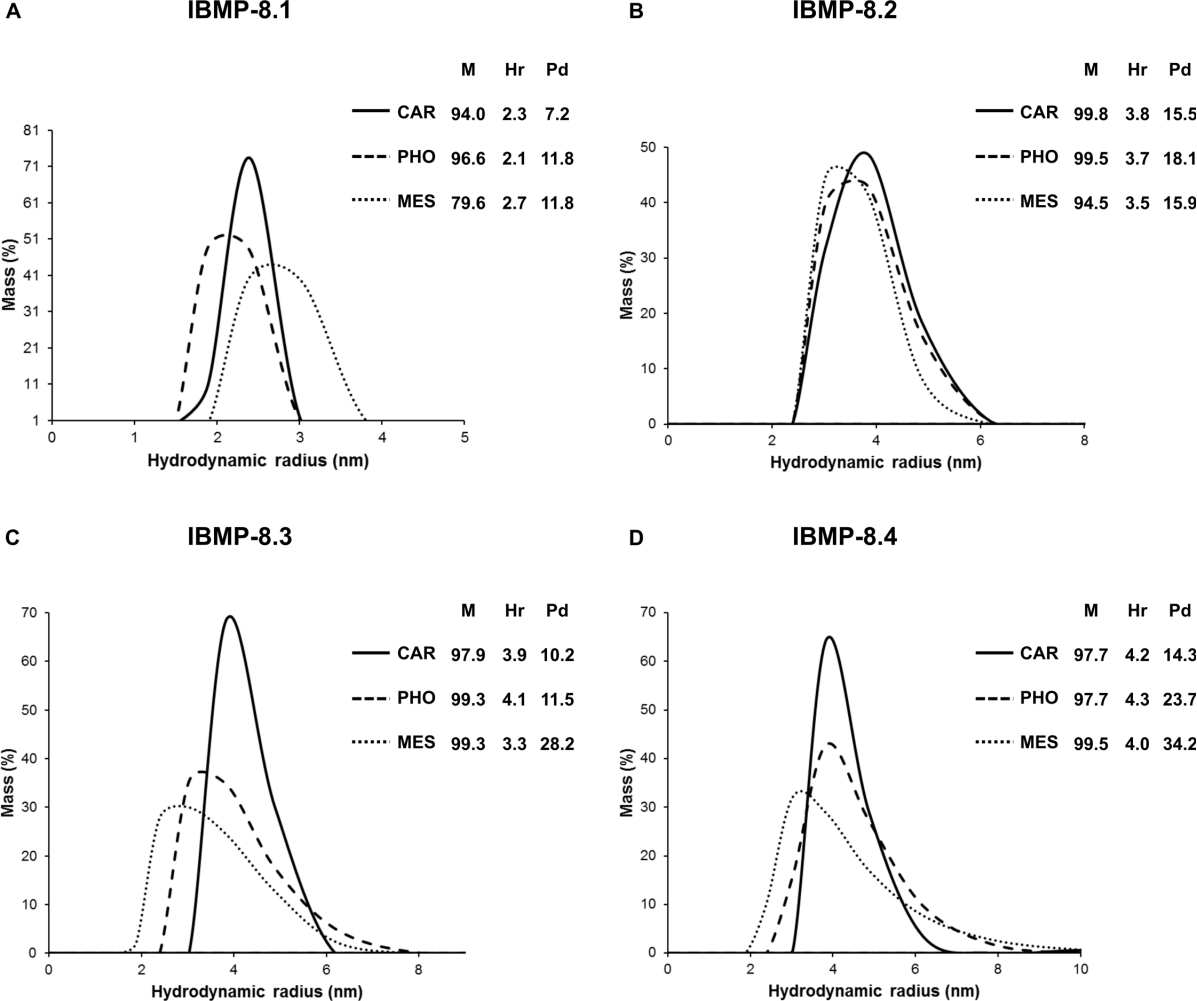
Dynamic light scattering measurements of IBMP recombinant chimeric proteins. Panel A) IBMP-8.1. Panel B) IBMP-8.2. Panel C) IBMP-8.3. Panel D) IBMP-8.4. M (% mass); Hr (Hydrodynamic radius); Pd (Polydispersity).

Circular Dichroism spectra of IBMP-8.1 and -8.3 proteins predominantly comprised random coil contributions as evidenced by neutral values between 215 and 240 nm and negative values at about 205 nm (Fig 2A and 2C) and no conformational changes were observed in those coil proteins (Fig 2A and 2B) upon solubilization in different buffering agents. DLS data indicate that IBMP-8.1 was monodisperse in all buffers tested (polydispersity < 20%), with best structural homogeneity found in 50 mM carbonate-bicarbonate pH 9.6 (Fig 3A). Moreover, when in 50 mM MES (pH 5.5) ~28% of the sample appeared to aggregate (Fig 3A, % Mass). In solution, analysis of IBMP-8.3 indicates that this protein can be influenced by the environment. In acidic pH, IBMP-8.3 is polydisperse (Pd > 20%) and basic pH triggers protein aggregation (Fig 3C).

Far-UV region circular dichroism spectra of IBMP-8.2 and -8.4 proteins displayed negative minimums at 208 and 220 nm, which are characteristic for proteins composed of α-helices (Fig 2B and 2D). Increase in alpha-helical content was observed for IBMP-8.3 and -8.4 protein upon solubilization in acidic pH (50 mM MES, pH 5.5) (Fig 2C and 2D). However, DLS analysis revealed that in acidic pH, IBMP-8.2 and -8.4 displayed more evident aggregation in 50 mM MES pH 5.5 and in 50 mM sodium phosphate pH 7.5 compared to 50 mM carbonate-bicarbonate pH 9.6. Hydrodynamic radii values varied from 2.3 to 2.7 for IBMP-8.1, 3.5 to 3.8 for IBMP-8.2, 3.3 to 4.1 for IBMP-8.3, and 4.0 to 4.3 for IBMP-8.4 (Fig 3), which are consistent with their molecular mass. Circular dichroism and dynamic light scattering data are available in S1 and S2 Tables, respectively.

### Assay performance

**Fig 4:**
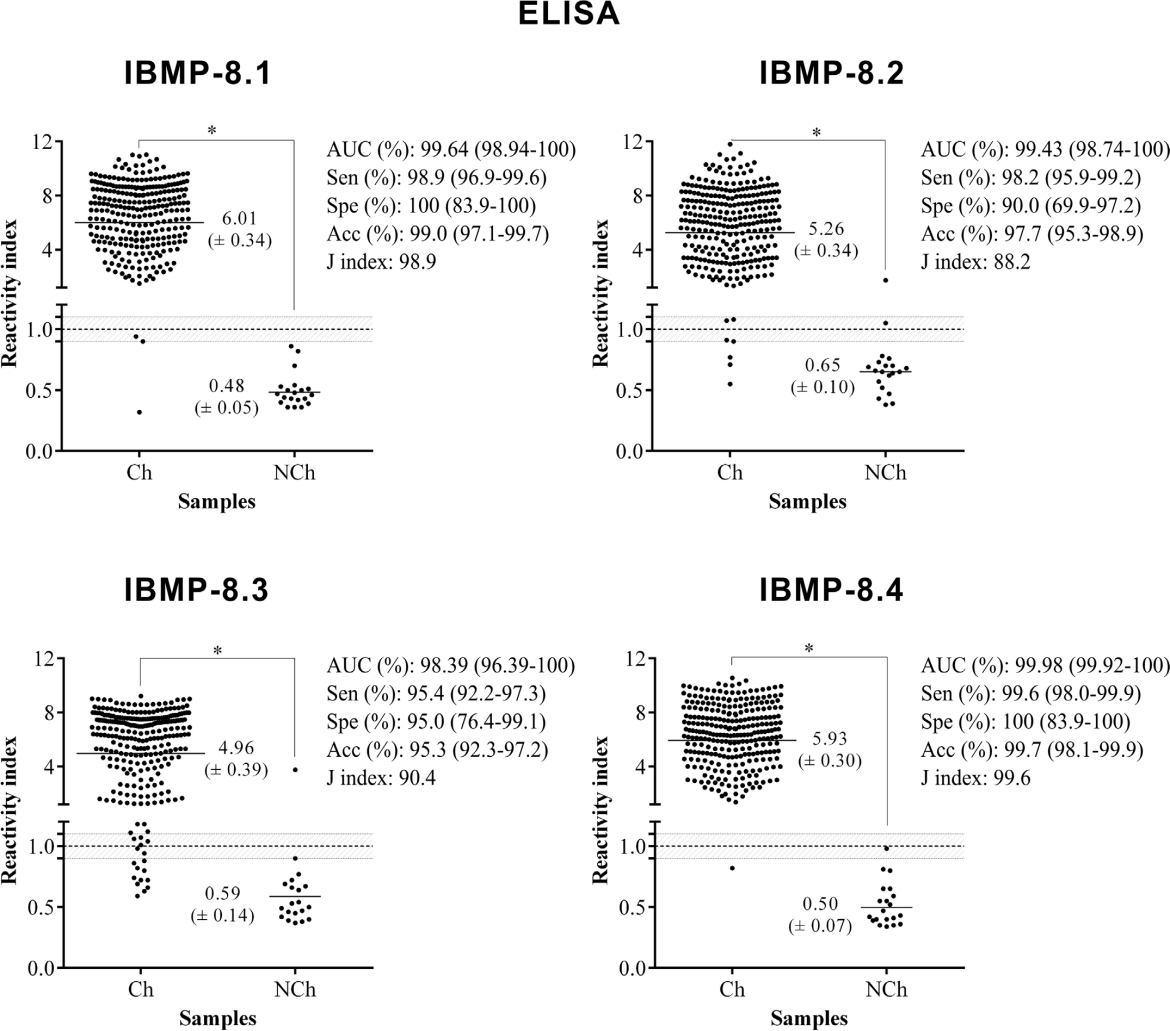
Anti-*Trypanosoma cruzi* IgG level in serum samples from chagasic (Ch) and non-chagasic (NCh) individuals assayed by ELISA. The cut-off value is 1.0 and shadowed area represents the grey-zone. Horizontal lines and numbers for each group of results represent the geometric means (± 95%CI).

**Fig 5:**
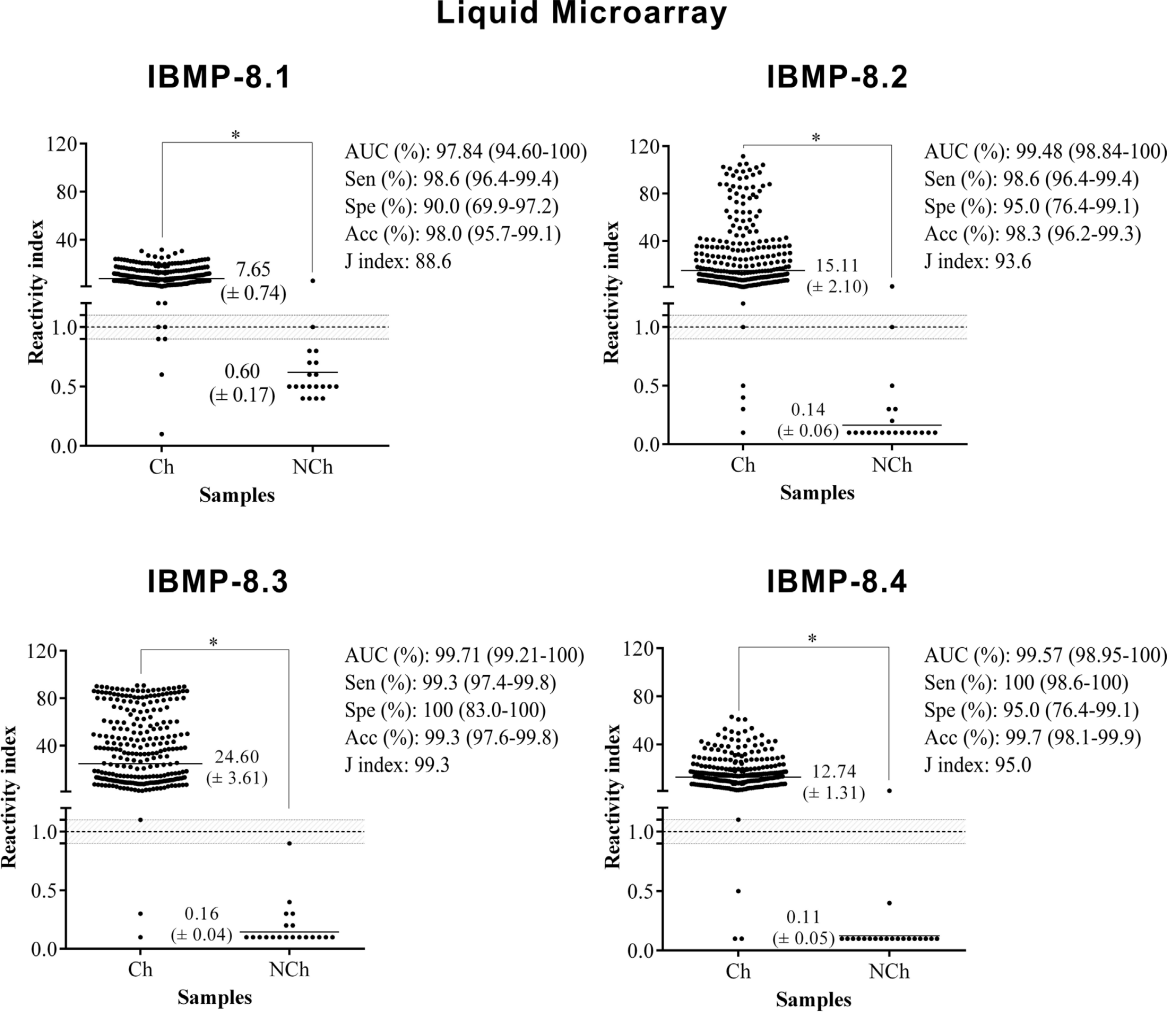
Anti-*Trypanosoma cruzi* IgG level in serum samples from chagasic (Ch) and non-chagasic (NCh) individuals assayed by liquid microarray. The cut-off value is 1.0 and shadowed area represents the grey-zone. Horizontal lines and numbers for each group of results represent the geometric means (± 95%CI).

Sera from 300 individuals living in an endemic area for CD previously tested for *T*. *cruzi* infection using two tests, as recommended by the Brazilian Health Ministry [6], were assayed by antigen-antibody detection tests using four recombinant chimeric proteins (data in S3 Table). Based on the total number of samples the area under ROC (AUC) for IBMP proteins varied from 0.9839 to 0.9998 (*p*<0.0001) as detected by ELISA and from 0.9784 to 0.9971 (*p*<0.0001) by LMA, demonstrating high diagnostic accuracy for all IBMP recombinant chimeric proteins (Figs 4 and 5). In the ELISA analyses, the sensitivity score yielded values of up to 98% for IBMP-8.1, -8.2 and -8.4 proteins and up 95% for IBMP-8.3. The global analysis of negative serum samples revealed that the lowest and highest negative values were presented by IBMP-8.2 (90%) and by IBMP-8.1 and -8.4 antigens (100%) (Fig 4). At 95% CI, the sensitivity and specificity scores showed no difference among the antigens. For positive samples, the IBMP-8.1 and -8.4 antigens displayed the highest RI followed by IBMP-8.2 and -8.3. Significant differences were evidenced between positive and negative samples considering the RI analysis (*p*<0.0001).

Similar results were obtained by LMA (Fig 5). With this methodology, sensitivity varied from 98.6% (IBMP-8.1 and -8.2) to 100% (IBMP-8.4) and specificity from 90% (IBMP-8.1) to 100% (IBMP-8.3). The highest RI was achieved by IBMP-8.3, followed by IBMP-8.2, -8.4, and -8.1. No significant difference was observed in the performance parameters when the IBMP antigens were evaluated by both ELISA and LMA methodologies, barring IBMP-8.3 sensitivity.

The agreement between the expected results provided by *T*. *cruzi* chimeric antigens varied from 95.3% for IBMP-8.3 to 99.7% for IBMP-8.4 in samples assayed by ELISA and 98.0% for IBMP-8.1 and 99.7% for IBMP-8.4 by LMA (Table 1). The high Cohen Kappa index (κ>0.81) of IBMP-8.1, -8.2 and -8.4 by ELISA and of IBMP-8.1 to -8.4 by LMA underline excellent agreement of test results, which showed almost perfect agreement with the reference tests. IBMP-8.3 in samples assayed by ELISA provided 14 discordant results, revealing substantial agreement (κ = 0.71) with reference tests.

**Table 1:** Strength of agreement of chimeric proteins for *Trypanosoma cruzi* IgG detection.

	Chimeras IBMP	Agreement to Reference Tests (%)	κ (95%CI)	Agreement
**EIA**	8.1	99.0	0.92 (0.84–1.01)	Almost perfect
	8.2	97.7	0.82 (0.70–0.95)	Almost perfect
	8.3	85.3	0.71 (0.56–0.86)	Substantial
	8.4	99.7	0.97 (0.92–1.03)	Almost perfect
**MIB**	8.1	98.0	0.85 (0.72–0.97)	Almost perfect
	8.2	98.3	0.87 (0.77–0.98)	Almost perfect
	8.3	99.3	0.95 (0.88–1.02)	Almost perfect
	8.4	99.7	0.97 (0.92–1.03)	Almost perfect

*κ*, Cohen’s Kappa coefficient; CI, confidence interval.

## Discussion

Here, we aimed to produce and validate four chimeric recombinant antigenic proteins for the precise detection of anti-*T*. *cruzi* antibodies in sera from individuals infected with *T*. *cruzi*. Selection of these antigens was based on literature descriptions considering their immunodominance, high affinity for antibodies, high signal in antigen-antibody methodologies, and ability to identify infected individuals living in different geographical areas [19–21,27,36–41]. Despite advances in the understanding of CD over the past 100 years since its discovery, the diagnosis of CD represents a challenge. In fact, no test has been shown to be sufficiently sensitive and specific to be designated as the sole screening assay for CD [6,10]. The diagnostic performance of several biochemically purified and recombinant/synthetic antigens have been assessed in the last few years as a way of improving the serodiagnosis of CD [27]. However, a considerable variation in the reproducibility, reliability, feasibility and cross-reactivity has been described. Recent investigations have shed light on potentially promising results obtained when chimeric recombinant proteins designated with selected amino acid sequences were assayed for CD diagnosis [20,21,40]. Although our data are preliminary at this point, our results provide high parameter performance values, indicating that IBMP chimeras may be used as antigenic matrix for CD diagnosis.

Ability of diagnostic tests to detect specific antibodies depends on the availability and spatial distribution of epitopes on the solid phase, which makes the understanding of the antigenic nature crucial to control the factors affecting the antigen-antibody reaction, such as pH, ionic strength, as well as the aggregate formation in the solvent in which it is immersed [42]. In fact, successful binding of antibodies to proteins has been correlated with hydrophilicity and exposure of epitopes [43,44]. Linearized epitopes allow that all individual fragments interact with polyclonal antisera, avoiding overlapping and competition. The IBMP antigens were subjected to Circular Dichroism evaluation in three buffering systems, which displayed predominance of random coil structures, despite little α helical structures observed in some antigens. Circular dichroism data also displayed that acidic pH provided an increase in the secondary-structure content for IBMP-8.3 and -8.4, which could lead to a potential difference in the diagnostic accuracy. So, on the basis of the above data, 50 mM carbonate-bicarbonate (pH 9.6) and 50 mM sodium phosphate (pH 7.5) buffers were recommended as sensitizing agents. However, DLS data displayed less polydispersity when IBMP antigens were dispersed in 50 mM carbonate-bicarbonate buffer (pH 9.6). This parameter measures the heterogeneity of sizes of molecules or particles dispersed in a solution. Aggregates leading to impairment of antigen-antibody detection caused by hidden or folded epitopes, hinder antigen accessibility, leading to a reduction in diagnostic performance. Therefore, we employed the 50 mM carbonate-bicarbonate (pH 9.6) as buffering system to sensitize the microplates.

Phase I studies are typically conducted using a group of seropositive individuals and a group of seronegative individuals, with an aim to assess whether the test is able to differentiate them [45,46]. In our study, the ELISA and LMA results using the IBMP recombinant chimeric proteins revealed that all antigens appropriately discriminated between negative and CD-positive samples (p<0.0001), as demonstrated by ROC curve analysis. A ROC curve analysis was chosen by permitting the analyst to fit test values and to achieve the best parameters to differentiate positive from negative samples. With this approach, ELISA-based diagnostic test achieved 99.6% sensitivity to IBMP-8.4 and 100% specificity, while the other proteins displayed a lower sensitivity and specificity. Regarding LMA, the test achieved maximum sensitivity to IBMP-8.4 and maximum specificity to IBMP-8.3. The differences in the specificities found by ELISA and LMA methods occurred due to the small number of negative samples used. However, despite this, we obtained high performance when all four IBMP recombinant chimeric proteins were used as antigen to detect specific anti-*T*. *cruzi* antibodies, suggesting that they are eligible to enter phase II studies.

## Supporting Information

S1 Table Circular Dichroism data. (XLSX)

S2 Table Dynamic Light Scattering data. (XLSX)

S3 Table Immunoassays (ELSIA and Liquid Microarray) data. (XLSX)
